# Preparation and Microbiological Evaluation of Amphiphilic Kanamycin-Lipoamino Acid Ion-Pairs

**DOI:** 10.3390/antibiotics3020216

**Published:** 2014-05-26

**Authors:** Rosario Pignatello, Antonio Leonardi, Giulio Petronio Petronio, Barbara Ruozi, Giovanni Puglisi, Pio Maria Furneri

**Affiliations:** 1 Department of Drug Sciences, University of Catania, Città Universitaria, viale A. Doria 6, I-95125 Catania, Italy; E-Mails: r.pignatello@unict.it (R.P.); antonio.leonardi@gmail.com (A.L.); puglisig@unict.it (G.P.); 2 NANO-*i*, Research Centre for Ocular Nanotechnology, Department of Drug Sciences, University of Catania, viale A. Doria 6, I-95125 Catania, Italy; 3 Department of Biomedical Sciences, University of Catania, via Androne 83, I-95124 Catania, Italy; E-Mail: gpetroniopetronio@gmail.com; 4 IRCCS San Raffaele Pisana, Via della Pisana 235, I-00163 Roma, Italy; 5 Pharmaceutical Technology, Te.Far.T.I. group, Department of Life Sciences, University of Modena and Reggio Emilia, via Campi 183, I-41100 Modena, Italy; E-Mail: barbara.ruozi@unimore.it

**Keywords:** coevaporates, physical mixtures, ion-pairs, lipoamino acids, amphiphilicity, DSC, PXDR, antibacterial activity, MIC, *E. coli*

## Abstract

Amphiphilic ion-pairs of kanamycin (KAN) were prepared by evaporation of a water-ethanol co-solution of KAN base and a lipoamino acid bearing a 12-carbon atoms alkyl side chain (LAA12), at different molar ratios. Infrared spectroscopy confirmed the structure of ion-pairs, while differential scanning calorimetry (DSC) and powder X-ray diffractometry (PXRD) studies supported the formation of new saline species with a different crystalline structure than the starting components. The solubility pattern shown in a range of both aqueous and organic solvents confirmed that the ion-pairs possess an amphiphilic character. The LAA12 counter-ion showed not to improve the antibacterial activity of KAN, suggesting that such chemical strategy is not able to favor the penetration of this drug inside the bacteria cells. Nevertheless, a slight improving, *i.e.*, a one-fold dilution, was observed in *E. coli*. The present study can also serve as the basis for a further evaluation of LAA ion-pairing of antibiotics, as a means to improve the loading of hydrophilic drugs into lipid-based nanocarriers.

## 1. Introduction

**Figure 1:**
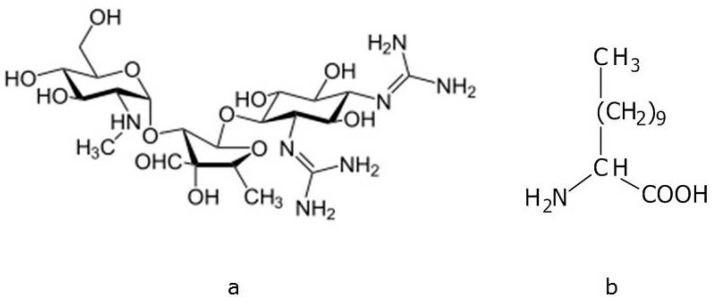
(**a**) Chemical structure of kanamycin A (KAN) (**b**) and LAA12.

Aminoglycoside antibiotics are bactericide agents extensively used in the clinical therapy of many infectious diseases [1]. Among them, kanamycin (KAN; Figure 1a) has been originally purified from the bacterium *Streptomyces kanamyceticus* and comprises three components: kanamycin A, the major component (usually designated as kanamycin), kanamycin B and C, as minor components [2]. Its irreversible binding to mRNA decodifying region of the 30S-subunit of the bacterial ribosome allows the inhibition of the protein synthesis [3,4].

KAN is effective against aerobic and facultative aerobic Gram-negative bacilli and some Gram-positive bacteria relevant to clinical infections, including staphylococci. Aminoglycoside penetration through the cell membrane is an aerobic, energy-dependent process: consequently, anaerobe bacteria are resistant to this class of antibiotics. Streptococci and enterococci are resistant to aminoglycosides because the drugs fail to penetrate through the cell wall of these bacteria [1,5,6]. Treatment of these organisms requires the co-administration with an inhibitor of cell wall synthesis, such as a penicillin or vancomycin [1]. A reduced cellular permeability can result in an insufficient (sub-active) concentration of the antibiotic in the target sites and a more successful inactivating activity of bacterial enzymes.

KAN has been submitted to extensive investigations looking for more active derivatives, as well as to preformulation studies aimed at obtaining more stable forms and effective dosage products. For instance, lipid conjugation was shown to ameliorate the *in vitro* activity [7] and to reduce KAN sensitivity to inactivating enzymes [8].

Aminoglycoside antibiotics are generally converted into a salt form during recovery and purification steps. KAN is commonly obtained as the water soluble mono- or disulfate salt. Other salts, such as the 3'-phosphate and the mono-, di- and tri-d-pantothenates have not reached clinical dignity.

Conversion to inorganic or simple organic salts can positively affect the pharmacokinetics of the antibiotic and also attenuate its typical oto- and nefrotoxicity [9], but may be unable to optimize their interaction with and uptake by bacterial cells. The hydrophobic ion-pairing (HIP) approach has been recently suggested as a chemical strategy to reversibly modify the properties of active compounds. Using HIP in molecules containing ionizable groups, polar counter-ions are stoichiometrically replaced with lipophilic ones. The resulting hydrophobic ion-pairs can improve drug permeability, allowing a better systemic (e.g., intestinal) adsorption and enhancing cellular uptake [10,11,12]. From a technological point of view, the amphiphilic features of ion-pairs can enhance the drug dissolution rate in organic solvents and the encapsulation and retention of hydrophilic compounds in drug delivery systems (DDS), and especially in liposomes and lipid-based nanocarriers [13,14,15,16]. Some antibacterial drugs have been also investigated by the HIP strategy [17,18].

We have recently undertaken a series of studies focused on the reversible modification of antibiotic molecules through the formation of organic amphiphilic ion-pairs, using lipoamino acids (LAA) as lipophilic counter-ions. LAA have become increasingly important because of their chemical simplicity and versatility, tenside-like surface activity, aggregation properties, and low toxicity pattern [19,20]. In particular, we have investigated some α-amino acids bearing in 2-position a saturated alkyl chain of different length (Figure 1b). Thanks to the presence of both an aliphatic chain and a polar amino acid head, LAA can impart amphiphilic properties (*membrane-like character*) to the drugs to which they are conjugated [21].

LAA have been then proposed to modulate or increase the interaction with and penetration through cell membranes and biological barriers of compounds with a poor biopharmaceutical profile [22,23]. Toth and co-workers have previously described β-lactam antibiotic derivatives covalently linked to LAA [24], with the aim at enhancing their gastro-intestinal adsorption. In those studies, oral administration of an ampicillin-LAA conjugate significantly improved the antibacterial activity [25]. More recently, a D-glucuronic acid-LAA derivative has been ion-paired with the antibiotic gentamicin, resulting in a better *in vivo* oral absorption [26].

The ability of drug-LAA complexes to interact with the biological membranes and to facilitate the drug penetration inside the target cells has been described by some of us through a covalent bond of drugs to LAA [27,28].

The aim of the present research project was instead to verify if the same possibility exists by forming reversible electrostatic complex between ionizable compounds and LAA. We have previously used the LAA ion-pairing strategy to produce amphiphilic derivatives of erythromycin (ERY) [29] and tobramyicin (TOB) [30]. The *in vitro* cell growth inhibitory activity profile of these ion-pairs was close to that of the parent drug (for ERY) or was even improved in terms of MIC (as seen with TOB) against different bacterial strains, both sensitive and resistant to these classes of antibiotics.

The presence of multiple amino groups makes KAN a hydrophilic polycation, with a reported dissociation constant (pka) of 7.2, an experimental LogP(o/w) of −6.7 [31] and a calculated LogD (pH 7.4) of −8.86 [32]. Such a hydrophilic nature can affect the low activity of this antibiotic, especially against anaerobic gram-negative bacteria [1]. Thus, in the present work we studied the preparation and *in vitro* microbiological profile of novel ion-pairs obtained using different KAN and LAA molar ratios.

## 2. Results and Discussion

This study was thus aimed to verify if an increased amphiphilicity, induced by ion-pairing KAN with a LAA moiety, will improve the antibacterial spectrum of activity of this drug.

A LAA moiety with a 12-carbon atom side alkyl chain (LAA12) (Figure 1b) was used in the present work. The KAN-LAA12 ion-pairs were prepared by reduced pressure evaporation of a water/ethanol co-solution of the drug (as the free base) and LAA12. To verify the effect of progressive ion-pairing of the four amine groups present in KAN molecule (Figure 1a), different drug to LAA initial molar ratios were tested (1:1, 1:2 or 1:4). FT-IR analysis was used to confirm the structure of the prepared ion-pairs, while powder X-ray diffractometry (PXRD) and differential scanning calorimetry (DSC) were employed to assess the formation of a new saline species with respect to the starting ingredients. The experimental data were compared with corresponding physical mixtures (PhMs) of KAN and LAA12, obtained by simple mechanical mixing of the two components of the ion-pairs, in the absence of any solvent.

As a preliminary biological assessment, the synthetized KAN-LAA12 ion-pairs and PhMs were tested *in vitro* against different bacterial strains to assess the effect of lipid moiety on the antibacterial activity of the drug.

The solubility of KAN-LAA12 ion-pairs in polar and apolar solvents of pharmaceutical interest is shown in Table 1. Compared to KAN free base, the ion-pairs were less soluble in water and in the phosphate buffer solution, but much more soluble in the tested organic solvents. Only ethyl acetate showed limited solvent properties for KAN-LAA ion-pairs.

**Table 1:** Solubility (mg/mL) of KAN-LAA coevaporates in different solvents at room temperature, compared to KAN base and KAN sulfate.

Solvent	KANbase	*KAN* *sulfate*	KAN-LAA12 1:1	KAN-LAA12 1:2	KAN-LAA12 1:4
Water	soluble *^a^*	*10–50*	7.5	7.5	7.5
Phosphate buffer (0.13 M, pH 7.4)	soluble	*n.a.*	5	7.5	15
Ethanol	slightly soluble	*insoluble ^b^*	10	15	>20
Acetone	slightly soluble	*n.a.*	2.5	7.5	>20
Ethyl acetate	insoluble	*n.a.*	1	2.5	2.5
Dichloromethane	insoluble	*insoluble*	5	15	>20

^a^ [33,34]; ^b^ [33]; *n.a.*: Not available data.

**Figure 2:**
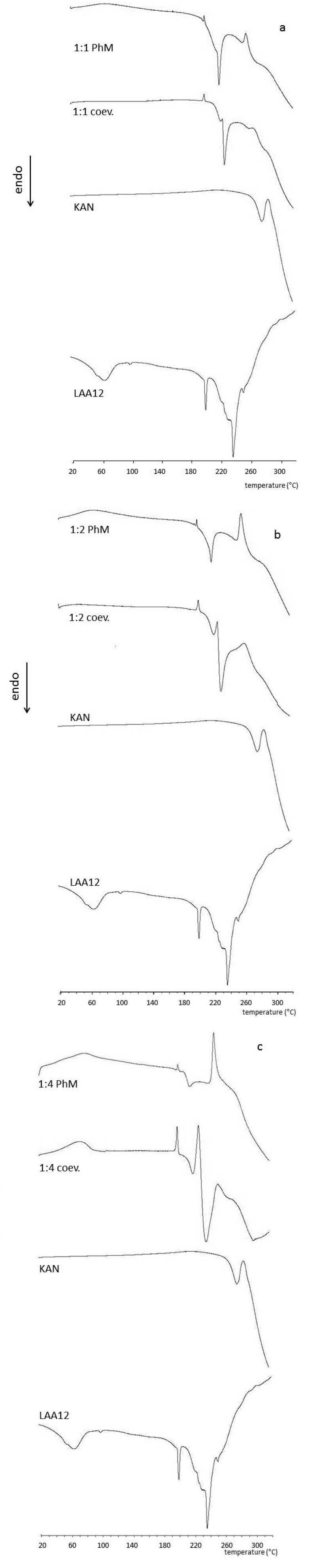
Differential scanning calorimetry (DSC) curves of KAN-LAA12 ion-pairs and physical mixtures (PhMs).

The physical state of KAN-LAA coevaporates and PhMs was investigated by various conventional techniques. The DSC curve of KAN A (free base) showed an endothermic peak at 268 °C, corresponding to its melting point (Figure 2a). Pure LAA12 instead showed strong endothermic melting peaks in the range between 220 and 240 °C (Figure 2a). The KAN-LAA12 ion-pairs showed a different DSC profile (Figure 2a–c), in which the signals of the two starting ingredients were not more visible, and a new, strong endothermic peak appeared around 210 °C, most probably due to the gradual melting of the formed ion-pair.

**Figure 3:**
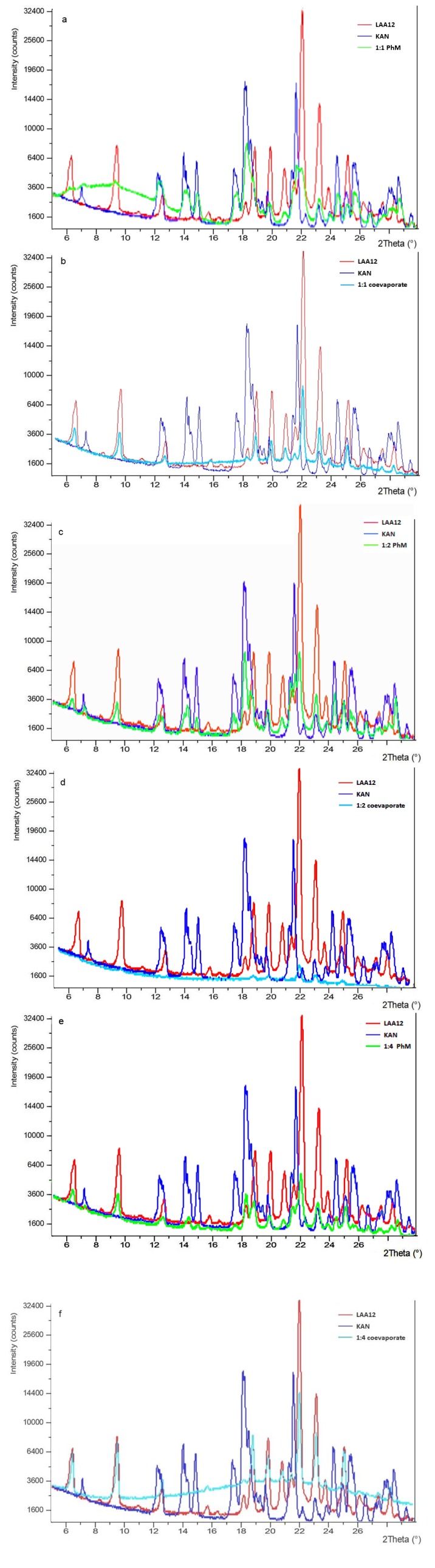
Power X-ray diffractometry (PXRD) profiles of PhMs (**a**, **c** and **e**) and coevaporates (**b**, **d** and **f**) between KAN and LAA12 prepared at 1:1, 1:2 or 1:4 molar ratio.

The PXRD spectra of the starting pure ingredients were compared with the PhMs and coevaporates obtained at different ratios. The spectrum of pure KAN base returned the following characteristic reflection described as 2θ in °, rel. intensities: ss = very strong, s = strong, m = medium, mw = medium-weak, w = weak: 7.1 (mw), 12.4 (m), 14.4 (s), 14.9 (s), 17.6 (s), 18.3 (ss), 19.7 (mw), 21.7 (ss), 22.4 (w), 23.2 (w), 24.0 (w), 24.5 (m), 25.1 (mw), 25.6 (m), 26.6 (mw), 27.4 (w), 27.6 (w), 28.4 (m), 28.7 (m), 29.6 (w) (Figure 3a). Also the XRD pattern of pure LAA12 showed intense and sharp peaks that prove the crystalline nature of this compound: 6.5 (mw), 9.6 (m), 12.7 (w), 18.2 (w), 18.9 (m), 20 (m), 20.9 (mw), 22.2 (ss), 23.4 (s), 23.9 (w), 25.3 (mw), 26.3 (w), 27.6 (w), 28.4 (w) (Figure 3a).

Considering the KAN-LAA12 1:1 molar ratio, the spectrum of the PhM was rich in signals corresponding to the profile of both drug and LAA crystals (in particular, the signal at ~21° and a complicate signal near 22.2°) (Figure 3a). This confirmed its nature of a “mixed” powder between two distinct ingredients, which did not form strong chemical interconnections, and thus no novel crystalline entity originated. The signals relative to KAN completely disappeared in the spectrum of the 1:1 coevaporate. Its diffractometric patterns were largely superimposable to that registered for the crystals of LAA12, even if a marked reduction in intensity can be observed (Figure 3b).

As regards the drug-LAA12 1:2 molar ratio, the spectrum of PhM showed both signals of the starting compounds, with a slight decrease of their relative intensity (Figure 3c). In a different way, the spectrum of the relative coevaporate described the formation of a new structure with evident loss of crystallinity, as a consequence of the molecular dispersion of KAN in the LAA matrix (Figure 3d). Peaks at 2θ values of 18.9°, 20°, 22.2°, 23.3° and 25.3°, attributable to the LAA12, were still recognizable but the very low intensity of these peaks is an ulterior proof of the drug dispersion occurred during the coevaporation process.

If the 1:2 coevaporate showed the diffractometric profile of an amorphous materials, the 1:4 coevaporate gave a diffractogram largely superimposable to that registered for LAA12 (Figure 3f), as the possible consequence of the excess of a “free” aliquot of this compound in the coevaporate. Also in this case, the intensities of the signals were less intense in the coevaporates compared to the LAA alone, indicating the formation of a more amorphous structure with respect to the starting components.

Results of FT-IR analysis are resumed in Table 2. The IR spectrum of pure KAN (free base) showed intense bands in the 1000−1265 cm^−1^ region, typical for carbohydrate absorptions. LAA12 spectrum was instead characterized by the carboxyl stretching signal, in the 1590–1720 cm^−1^ range. Nevertheless, KAN and LAA12 spectra showed common absorption bands, such as in the 3500–3350 cm^−1^ region, at 1660 and at 1000–1200 cm^−1^. Also, this analysis confirmed the formation of new saline species between KAN and LAA12, with the appearance of new signals (evidenced in red in Table 2) that the spectra of the starting components. In particular, the shift at lower fields (1215–1213 cm^−1^) of the C-O stretching peak of the LAA12 moiety, evidenced the occurrence of some ionic interactions. The corresponding physical mixtures also showed the original bands of pure drug and LAA12 but, in these cases, and compared with the co-evaporates, the signals have stronger intensities. It is noteworthy that the presence of strong bands at 1215 cm^−1^ suggested the development of ionic interactions between the LAA carboxyl function and the drug amine groups, even after the simple mechanical dry mixing at room temperature. The same behavior had been observed for the LAA coevaporates with ERY and TOB [29,30].

**Table 2:** FT-IR spectroscopy data of pure KAN (in red) and LAA12 (in black) and of their coevaporates and PhM at different molar ratios. Spectra were taken as nujol mulls; values are expressed in cm^−1^. Data in blue indicate signals not present in the spectra of the starting compounds.

LAA12	KAN	KAN/LAA12					
		1:1 coev.	1:1 PhM	1:2 coev.	1:2 PhM	1:4 coev.	1:4 PhM
	3500–3350	3350	3500–3350	3350–3215	3350–3215	3350	3500–3350
3400							
						1750	
						1730	
1720			1720	1720	1715	1715	
1660	1660	1660	1660	1660	1660	1665	1665
	1655	1655				1655	
1637		1637	1637	1635	1637		1635
	1630		1630				
1615		1618		1615	1610	1615	1615
	1592		1595				
1583		1583		1583	1580	1585	1585
	1525		1525		1525		
1510		1505					1513
1340							
1310		1310		1310		1310	1310
					1290		
	1265				1260		
1250							
1230			1230		1230		
	1225						
		1215	1215	1215		1213	
1194				1194	1200		
1160							
	1154	1154	1150	1154	1154	1156	1156
1093							
	1031	1031	1040	1031	1031	1031	1025

The *in vitro* antibacterial activities of the synthesized coevaporates were compared with that of the parent drug by the MIC method (Table 3). Both KAN susceptible and unsusceptible strains were tested for the purpose. As a further comparison, the starting LAA12 and the various KAN-LA112 PhMs were included in the assay.

**Table 3:** Minimum inhibitory concentrations (MIC, µg/mL) of KAN-LAA coevaporates and PhMs, compared to pure LAA12 and KAN sulfate. MIC values were calculated based on the actual KAN concentration in each sample (except for pure LAA12 sample). The reported results are the mean of 12 replications (six times with each formulation and six additional times on a different day).

Compound	*E. coli* * ATCC 25922	*E. faecalis* * ATCC 29212	*S. pneumoniae* (No. 5)	*L. fermentum* (No. 2)
KAN sulfate	4	≥16	≥16	≥16
LAA12	growth	growth	growth	growth
KAN-LAA12, 1:1	4	≥16	≥16	≥16
1:1 PhM	4	≥16	≥16	≥16
KAN-LAA12, 1:2	**2**	≥16	≥16	≥16
1:2 PhM	4	≥16	≥16	≥16
KAN-LAA12, 1:4	**2**	≥16	≥16	≥16
1:4 PhM	4	≥16	≥16	≥16

* Quality control strains according to Clinical and Laboratory Standard Institute [35].

Pure KAN (as the free base) showed the known activity profile, with a MIC value of 4 µg/mL against *E. coli*. Despite the higher MIC values (MIC >16 µg/mL) against *E. faecalis* the activity of the free drug was within the published quality control range [35]. The absence of any free drug activity against *S. pneumoniae* and *Lactobacillus* spp. was expected. As previously demonstrated, the pure lipoamino acid counter-moiety (LAA12) was devoid of any antibacterial activity against all the tested strains [29].

The antibacterial activity of KAN-LAA12 ion-pairs was close to that of the parent drug, and even one order higher with the 1:2 and 1:4 molar ratios. The corresponding PhMs gave an identical growth inhibitory profile than the free drug.

## 3. Experimental

KAN A free base was purchased from Santa Cruz Biotechnology Inc. (Dallas, TX, USA). HPLC-grade water and absolute ethanol were purchased from Merck (Darmstadt, Germany). The 2-amino-D,L-dodecanoic acid (LAA12) was synthesised in our lab from diethyl acetamido malonate and 1-bromodecane, according to a published procedure [36].

FT-IR spectra were registered in nujol with a Perkin-Elmer 1600 spectrophotometer. PXRD data were collected with a Philips X’Pert Pro X-ray diffraction system (Eindhoven, The Netherlands) (available at the Centro Interdipartimentale Grandi Strumenti di Modena e Reggio Emilia—CIGS) equipped with a PANanalytical solid state detector, operating in reflection mode, with a CuKα radiation (without monochromator: λ is a “mixing” between λ_Kα1_ = 1.540598 and λ_Kα2_ = 1.544426; Kβ radiation was removed by Ni foil). X-ray data were collected over a range 10 < 2θ < 30 at room temperature; the scan-rate was set at 0.007°/s. DSC experiments were performed with a Mettler DSC12E calorimeter. The detection system consisted of a Mettler Pt 100 sensor, with a thermometric sensitivity of 56 µV/°C, a calorimetric sensitivity of about 3 µV/mW and a noise less than 60 nV (<1 mV). Each DSC scan showed an accuracy of ±0.4 °C and reproducibility and resolution of 0.1 °C. Samples (3–10 mg) were sealed in a 40-µL aluminum pan, using an empty pan as reference. Each sample was analyzed from 30–320 °C, at a heating rate of 5 °C/min.

### 3.1. Ion-Pair Preparation

KAN-LAA12 ion-pairs were prepared by the evaporation of a co-solution of the two components. KAN base (0.3 mmoles) was dissolved in water, while the LAA12 (0.3, 0.6 or 1.2 mmoles) was slowly dissolved under magnetic stirring at room temperature in absolute ethanol. The obtained solutions were mixed for about 4 h at 40 °C and then at room temperature overnight. Ethanol and part of the water were removed off under high vacuum at an external maximal temperature of 40 °C. To remove the residual water, the sample was frozen in liquid nitrogen and lyophilized overnight (Modulyo freeze-dryer system; Edwards, Trezzano sul Naviglio, Italy). The resulting fluffy white powders were stored in tight closed glass vials at 4 ± 1 °C until use.

### 3.2. Physical Mixtures

KAN and the LAA12 were prior kept overnight at about 40 °C under high vacuum in a Büchi glass oven. KAN-LAA12 PhMs were prepared by triturating the two ingredients, at the same 1:1, 1:2, or 1:4 molar ratios, in a porcelain mortar for 30 min. These mixtures were also stored in a refrigerator in tight closed glass vials.

### 3.3. Solubility Determination

The solubility profile of the prepared coevaporates in a range of pharmaceutically related solvents (water; 0.13 M phosphate buffer solution, pH 7.4; ethanol; acetone; ethyl acetate; dichloromethane) was measured at room temperature. To a known volume of each solvent (about 2 mL) small amounts of KAN (free base) or KAN-LAA12 ion-pairs were progressively added. The mixture was vortex-mixed for 3 min and analysed by turbidimetry (Shimadzu UV-1601). The first detection of a measurable absorbance at 650 nm was considered as the solubility limit of the test material. Results are reported in Table 1.

### 3.4. Bacterial Strains

*Escherichia coli* ATCC 25922, *Enterococcus faecalis* ATCC 29212, and freshly isolates strains of *Streptococcus pneumoniae* (No. 5) and *Lactobacillus fermentum* (No. 2).

### 3.5. Susceptibility Test Procedure

The antimicrobial activity of KAN-LAA ion-pairs was investigated in comparison with that of the corresponding PhMs and the free drug, using the MIC values calculated by the standard broth microdilution assay [35]. Each ion-pair or PhM suspension was added in order to obtain an equivalent drug concentration with respect to that of the free drug solution. Mueller-Hinton broth was replaced by Iso-Sensitest broth (Oxoid, Basingstoke, UK) as previously described [37]. Briefly, an original solution in DMSO at 5.12 mg/mL of each KAN-LAA12 ion-pair and KAN was used as stock solution and then diluted 1/10 in Iso-Sensitest broth to obtain a working dilution of 512 µg/mL. The further dilutions were obtained as proposed by the Clinical Laboratory Standards Institute [36]. A total of 11 concentrations of each sample were prepared. A suspension of organisms (1 µL of a suspension containing 10^7^ CFU/mL) was added to each well. A positive control (growth) consisting of organisms in broth, a negative control (sterility) consisting of uninoculated broth, drug control consisting of broth containing the highest concentrations of KAN, and pure LAA12 (at concentrations 1, 10, and 100 times higher than those used throughout the experiments) were included for each bacterial strain tested. Plates were sealed with a transparent acetate foil and incubated at 37 °C under atmospheric conditions for up to 18 h.

## 4. Conclusions

This study was aimed at testing whether the conjugation with an LAA promoiety can improve the amphiphilic character of KAN. For this antibiotic, an enhanced penetration in bacteria cells could be the mean for enlarging its spectrum of activity, for instance against anaerobic microorganisms. Solubility data suggest that KAN-LAA ion-pairs have an amphiphilic character, which can help both in the formulation of controlled drug delivery carriers and for modifying the absorption profile of the drug after systemic administration.

The formation and structure of ion-pairs between the drug and the LAA moiety were confirmed by calorimetric and spectroscopic techniques. The different nature of the ion-pairs with respect to the corresponding PhMs was substantiated by PXRD studies.

The microbiological results confirmed that forming KAN ion-pairs with LAA12 did not distress the *in vitro* antibacterial potency of the drug, although no improvement in its spectrum of activity was achieved.

As a side aim of this study, it is however conceivable that the increased lipophilic character of KAN-LAA ion-pairs would positively affect drug encapsulation and retention in lipid-based carrier systems, such as liposomes and lipid nanoparticles. Several reports in the literature support the idea of optimizing the absorption or fate of compounds by combining a prodrug strategy and lipid nanocarriers [38,39,40,41]. This technological potentiality is going to be exploited in a separate study.

## References

[1] Gilbert D.N., Leggett J.E., Mandell G.L., Bennett J.E., Dolin R. (2010). Aminoglycosides. Principles and Practice of Infectious Diseases.

[2] EMEA The European Agency for the Evaluation of Medicinal Products—Veterinary Medicines and Inspections. Committee for Veterinary Medicinal Products—Kanamycin: Summary Report 1999 EMEA/MRL/514/98-FINAL. http://www.ema.europa.eu/docs/en_GB/document_library/Maximum_Residue_Limits_-_Report/2009/11/WC500014535.pdf.

[3] Lynch S.R., Puglisi J.D. (2001). Structure of a eukaryotic decoding region A-site RNA. J. Mol. Biol..

[4] Lynch S.R., Puglisi J.D. (2001). Structural origins of aminoglycoside specificity for prokaryotic ribosomes. J. Mol. Biol..

[5] Shaw K.J., Rather P.N., Hare R.S., Miller G.H. (1993). Molecular genetics of aminoglycoside resistance genes and familial relationships of the aminoglycoside-modifying enzymes. Microbiol. Rev..

[6] Pagès J.M., James C.E., Winterhalter M. (2008). The porin and the permeating antibiotic: A selective diffusion barrier in Gram-negative bacteria. Nat. Rev. Microbiol..

[7] Bera S., Zhanel G.G., Schweizer F. (2010). Antibacterial activity of guanidinylated neomycin B- and kanamycin A-derived amphiphilic lipid conjugates. J. Antimicrob. Chemother..

[8] Yang L., Ye X.S. (2010). Development of aminoglycoside antibiotics effective against resistant bacterial strains. Curr. Top. Med. Chem..

[9] Takemi K., Masayuki Y., Tetsutaro N., Kazuko M., Takashi T., Shigeharu I. (1980). An Aminoglycosidic Antibiotic Salt. EP.

[10] Neubert R. (1989). Ion-pair transport across membranes. Pharm. Res..

[11] Anderberg E.K., Lindmark T., Artursson P. (1993). Sodium caprate elicits dilatations in human intestinal tight junctions and enhances drug absorption by the paracellular route. Pharm. Res..

[12] Dai W.G., Dong L.C. (2007). Characterization of physiochemical and biological properties of an insulin/lauryl sulfate complex formed by hydrophobic ion-pairing. Int. J. Pharm..

[13] Gaudana R., Parenky A., Vaishya R., Samanta S.K., Mitra A.K. (2011). Development and characterization of nanoparticulate formulation of a water soluble prodrug of dexamethasone by HIP complexation. J. Microencapsul..

[14] Sun S., Liang N., Kawashima Y., Xia D., Cui F. (2011). Hydrophobic ion-pairing of an insulin-sodium deoxycholate complex for oral delivery of insulin. Int. J. Nanomed..

[15] Gallarate M., Chirio D., Bussano R., Peira E., Battaglia L., Baratta F., Trotta M. (2013). Development of O/W nanoemulsions for ophthalmic administration of timolol. Int. J. Pharm..

[16] Zhang T., Zheng Y., Peng Q., Cao X., Gong T., Zhang Z. (2013). A novel submicron emulsion system loaded with vincristine—Oleic acid ion-pair complex with improved anticancer effect: *In vitro* and *in vivo* studies. Int. J. Nanomed..

[17] Matschiner S., Neubert R., Wohlrab W., Matschiner F. (1996). Influence of ion-pairing on *ex vivo* penetration of erythromycin into sebaceous follicles. Skin Pharmacol..

[18] Dave R.N., Joshi H.M., Venugopalan V.P. (2011). Novel biocatalytic polymer-based antimicrobial coatings as potential ureteral biomaterial: Preparation and *in vitro* performance evaluation. Antimicrob. Agents Chemother..

[19] Infante M.R., Pinazo A., Seguer J. (1997). Non-conventional surfactants from amino acids and glycolipids: Structure, preparation and properties. Colloids Surf. A Physicochem. Eng. Asp..

[20] Clapés P., Infante M.R. (2002). Amino acid-based surfactants: Enzymatic synthesis, properties and potential applications. Biocatal. Biotransform..

[21] Toth I. (1994). A novel chemical approach to drug delivery: Lipidic amino acid conjugates. J. Drug Target..

[22] Pignatello R., Guccione S., Castelli F., Sarpietro M.G., Giurato L., Lombardo M., Puglisi G., Toth I. (2006). Enhancement of drug affinity for cell membranes by conjugation with lipoamino acids II. Experimental and computational evidence using biomembrane models. Int. J. Pharm..

[23] Sarpietro M.G., Micieli D., Pignatello R., Liang M.T., Toth I., Castelli F. (2008). Effect of variation in the chain length and number in modulating the interaction of an immunogenic lipopeptide with biomembrane models. Thermochim. Acta.

[24] Valkò K., Toth I., Ward P., Slegel P., Gibbons W.A. (1992). Lipidic peptides. XI. Quantitative structure-activity relationship of a series of lipidic amino acid conjugates of β-lactam antibiotics. Int. J. Pharm..

[25] Toth I., Hughes R.A., Ward P., McColm A.M., Cox D.M., Anderson G.J., Gibbons W.A. (1991). Fatty peptides. VI. Penicillin and cephalosporin esters with increased lipophilic character. Int. J. Pharm..

[26] Ross B.P., DeCruz S.E., Lynch T.B., Davis-Goff K., Toth I. (2004). Design, synthesis, and evaluation of a liposaccharide drug delivery agent: Application to the gastrointestinal absorption of gentamicin. J. Med. Chem..

[27] Pignatello R., Pantò V., Salmaso S., Bersani S., Pistarà V., Kepe V., Barrio J.R., Puglisi G. (2008). Flurbiprofen derivatives in Alzheimer’s disease: Synthesis, pharmacokinetic and biological assessment of lipoamino acid prodrugs. Bioconjug. Chem..

[28] Pignatello R., Paolino D., Pantò V., Pistarà V., Calvagno M.G., Russo D., Puglisi G., Fresta M. (2009). Lipoamino acid prodrugs of paclitaxel: Synthesis and cytotoxicity evaluation on human anaplastic thyroid carcinoma cells. Curr. Cancer Drug Targets.

[29] Pignatello R., Mangiafico A., Ruozi B., Puglisi G., Furneri P.M. (2011). Amphiphilic erythromycin-lipoamino acid ion-pairs: Characterization and *in vitro* microbiological evaluation. AAPS PharmSciTech.

[30] Pignatello R., Mangiafico A., Basile L., Ruozi B., Furneri P.M. (2011). Amphiphilic ion-pairs of tobramycin with lipoamino acids. Eur. J. Med. Chem..

[31] VSDB: Veterinary Substances DataBase at University of Hertfordshire Kanamycin Environmental Fate—Ecotoxicology—Human Health [updated 2013 May 26]. http://sitem.herts.ac.uk/aeru/vsdb/Reports/1921.htm/.

[32] Chemspider by Royal Society of Chemistry Kanamycin. http://www.chemspider.com/Chemical-Structure.5810.html/.

[33] Cron M.J., Fardig O.B., Johnson D.L., Palermiti F.M., Schmitz H., Hooper I.R. (1958). The basic and clinical research of the new antibiotic, kanamycin. Ann. NY Acad. Sci..

[34] National Center for Biotechnology Information Kanamycin—Compound Summary. PubChem Compound [updated 2005 June 24]. http://pubchem.ncbi.nlm.nih.gov/summary/summary.cgi?cid=6032#x27/.

[35] Clinical and Laboratory Standards Institute (2012). Performance Standards for Antimicrobial Susceptibility Testing.

[36] Gibbons W.A., Hughes R.A., Charalambous M., Christodoulou M., Szeto A., Aulabaugh A.E., Mascagni P., Toth I., Lipidic peptides I. (1990). Synthesis, resolution and structural elucidation of lipidic amino acids and their homo- and hetero-oligomers. Liebigs Ann. Chem..

[37] Puglisi G., Fresta M., Mazzone G., Furneri P.M., Tempera G. (1995). Formulation parameters of fluoroquinolones-loaded liposomes and *in vitro* antimicrobial activity. Int. J. Pharm..

[38] Gabizon A.A., Tzemach D., Horowitz A.T., Shmeeda H., Yeh J., Zalipsky S. (2006). Reduced toxicity and superior therapeutic activity of a mitomycin C lipid-based prodrug incorporated in pegylated liposomes. Clin. Cancer Res..

[39] Stancampiano A.H.S., Puglisi G., Pignatello R. (2008). Effect of lipophilicity of dispersed drugs on the physicochemical and technological properties of solid lipid nanoparticles. Open Drug Deliv. J..

[40] Wang J.J., Liu K.S., Sung K.C., Tsai C.Y., Fang J.Y. (2009). Skin permeation of buprenorphine and its ester prodrugs from lipid nanoparticles: Lipid emulsion, nanostructured lipid carriers and solid lipid nanoparticles. J. Microencapsul..

[41] Kuznetsova N.R., Sevrin C., Lespineux D., Bovin N.V., Vodovozova E.L., Mészáros T., Szebeni J., Grandfils C. (2012). Hemocompatibility of liposomes loaded with lipophilic prodrugs of methotrexate and melphalan in the lipid bilayer. J. Control. Release.

